# Comparison of two human organoid models of lung and intestinal inflammation reveals Toll‐like receptor signalling activation and monocyte recruitment

**DOI:** 10.1002/cti2.1131

**Published:** 2020-05-05

**Authors:** Shyam Sushama Jose, Marco De Zuani, Federico Tidu, Marcela Hortová Kohoutková, Lucia Pazzagli, Giancarlo Forte, Roberta Spaccapelo, Teresa Zelante, Jan Frič

**Affiliations:** ^1^ International Clinical Research Center St. Anne's University Hospital Brno Brno Czech Republic; ^2^ Department of Biology Faculty of Medicine Masaryk University Brno Czech Republic; ^3^ Department of Experimental Medicine and University Research Center for Functional Genomic (C.U.R.Ge.F) University of Perugia Perugia Italy; ^4^ Institute of Hematology and Blood Transfusion Prague Czech Republic

**Keywords:** immune response, infection, leucocyte migration, tissue organoids, Toll‐like receptors

## Abstract

**Objectives:**

The activation of immune responses in mucosal tissues is a key factor for the development and sustainment of several pathologies including infectious diseases and autoimmune diseases. However, translational research and personalised medicine struggle to advance because of the lack of suitable preclinical models that successfully mimic the complexity of human tissues without relying on *in vivo* mouse models. Here, we propose two *in vitro* human 3D tissue models, deprived of any resident leucocytes, to model mucosal tissue inflammatory processes.

**Methods:**

We developed human 3D lung and intestinal organoids differentiated from induced pluripotent stem cells to model mucosal tissues. We then compared their response to a panel of microbial ligands and investigated their ability to attract and host human primary monocytes.

**Results:**

Mature lung and intestinal organoids comprised epithelial (EpCAM^+^) and mesenchymal (CD73^+^) cells which responded to Toll‐like receptor stimulation by releasing pro‐inflammatory cytokines and expressing tissue inflammatory markers including MMP9, COX2 and CRP. When added to the organoid culture, primary human monocytes migrated towards the organoids and began to differentiate to an ‘intermediate‐like’ phenotype characterised by increased levels of CD14 and CD16.

**Conclusion:**

We show that human mucosal organoids exhibit proper immune functions and successfully mimic an immunocompetent tissue microenvironment able to host patient‐derived immune cells. Our experimental set‐up provides a novel tool to tackle the complexity of immune responses in mucosal tissues which can be tailored to different human pathologies.

## Graphical abstract

We propose a novel tool to study the interactions between human mucosal tissues and infiltrating monocytes. Taking advantage of two models of human‐induced pluripotent stem cell‐derived lung and gut organoids, we compared their response to a panel of microbial ligands and their ability to host human primary monocytes. We show that both the organoids – even in the absence of tissue‐resident immune cells – successfully mimicked an immunocompetent environment able to respond to microbial insults and to promote the differentiation of patient‐derived immune cells.

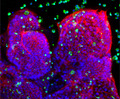

## Introduction

Mucosal tissues, especially the lungs and gut, provide a large surface area for host–microbe interaction and act as the primary defence against potential pathogens by providing a physical barrier, secreting immune molecules and mediating specific cellular immunity. Tightly orchestrated interactions of innate and adaptive immune cells^1^ with the cells forming the mucosal tissue result in the release of cytokines, chemokines and other molecules – such as mucus and defensins, important for the maintenance of tissue homeostasis.^2^, ^3^

The epithelial fraction of mucosal tissues acts as the primary source of cytokines mediating the recruitment of immune cells both under steady‐state conditions and during infection.^4^, ^5^ Due to the cytokine milieu secreted by mucosal tissues, they are host to multiple tissue‐resident immune cell subsets.^6^ During infection, the intense recruitment of immune cells – including neutrophils and monocytes – increases the cellular complexity of these anatomical districts.^7^, ^8^ Recent studies show that monocytes participate in steady‐state surveillance of both lung and intestine, complementary to tissue‐resident macrophages, while differentiating into macrophages or dendritic cells only during inflammatory stimulation.^9^, ^10^, ^11^ Moreover, mucosal tissue‐resident macrophages are constantly replenished from locally differentiated monocytes.^12^ However, the exact mechanisms underlying the complex immune response of the mucosal microenvironment remain controversial.

Although the role of hematopoietic cells in the immune response of mucosal tissues has been intensively researched, few studies assess the specific role of nonhematopoietic mucosal cells in the regulation of immune responses to infections. Traditional experimental animal models often do not allow the study of specific tissue responses because of the presence of resident (and nonresident) immune cells and the difficulty of depleting the animal's immune system without affecting its development. Similarly, *ex vivo* human tissue samples are difficult to obtain – especially from healthy donor biopsies – and in any case still contain the existing resident population of immune cells (including tissue‐resident macrophages and dendritic cells). It is important to note that complex inflammatory pathologies such as inflammatory bowel disease (IBD) or idiopathic pulmonary fibrosis (IPF) often result in altered PRR expression and downstream cytokine secretion by the epithelial cells, leading to dysregulated leucocyte activation and migration.^13^, ^14^ On the one side, *in vitro* 2D cell cultures lack the intrinsic cellular complexity and three‐dimensional structure of the tissues and are thus unable to recapitulate a complete inflammatory microenvironment.^15^

In recent years, the use of murine and human 3‐dimensional (3D) *in vitro* models has increased as a result of the growing number of differentiation protocols available and the precise characterisation of these tissue models.^16^ More specifically, 3D tissue organoids aim to recreate the morphology, structural complexity and primitive functions of murine and human organs, allowing us to study *in vitro* pared‐down versions of complex environments.^17^, ^18^

Lung organoids (LOs) and intestinal organoids (IOs) have been used to study host–microbe interactions including those of the intestine and *Helicobacter pylori* and of lung tissues and airborne pathogens.^19^, ^20^ Microinjection of *H. pylori* into the lumen of IOs results in the expression of several chemokines and in the induction of NF‐κB‐driven inflammatory responses against the pathogen, recapitulating the hallmarks of *H. pylori*‐associated gastric ulcers.^21^ Similarly, the injection of *Salmonella typhimurium* into the luminal cavity of murine adult stem cell‐derived IOs increases transcription of pro‐inflammatory cytokines including IL‐1β, TNF and IL‐8.^22^ A similar result was obtained by microinjecting *S. typhimurium* into human‐induced pluripotent stem cell (iPSC)‐derived IOs.^23^ Furthermore, Hill *et al*.^24^ demonstrated that the epithelial layer of human IOs can establish a stable host–microbe symbiosis characterised by increased antimicrobial peptide production, sustained maturation of the mucus layer and improved barrier function. These findings overall indicate that the use of human 3D organoids represents a valid and more precise alternative to *in vivo* and other *in vitro* models to characterise the immune response at mucosal sites.

In this study, we use two well‐established models of human iPSC‐derived lung and intestinal organoids to ask whether mucosal organoids can be used to model tissue inflammation and innate immune cell interactions.

## RESULTS

### Human iPSC‐derived lung and intestinal organoids resemble mucosal tissues and express functional Toll‐like receptors

**Figure 1:**
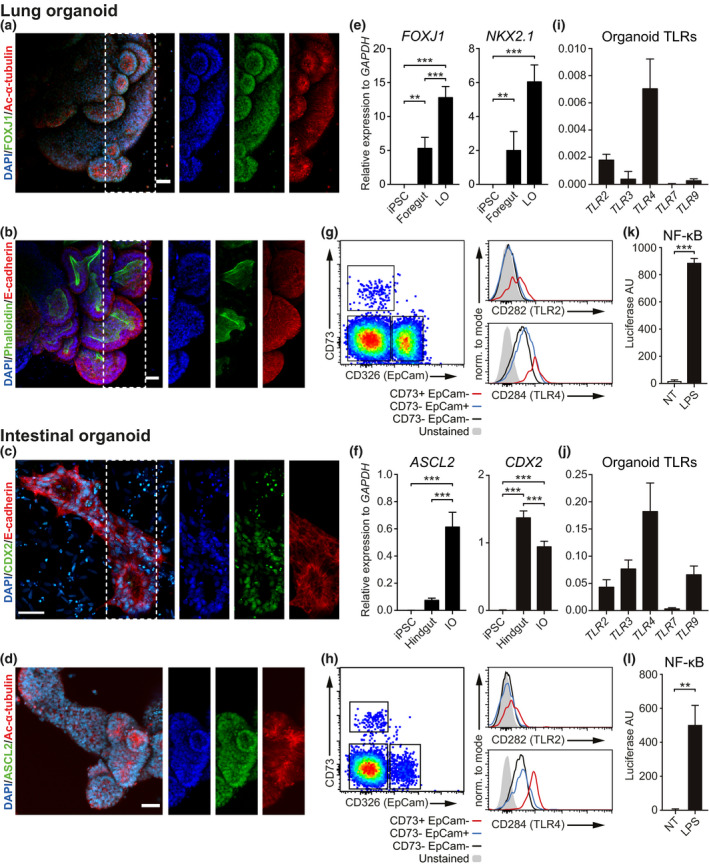
Human lung and intestinal organoids resemble mucosal tissues and express functional Toll‐like receptors (TLRs). Immunofluorescent labelling of human organoids shows tissue polarity and the expression of tissue‐specific transcription factors (TFs). Lung organoids expressed FOXJ1 TF and showed polarised localisation of acetyl‐α‐tubulin, E‐cadherin and phalloidin, suggesting highly self‐organised structures **(a, b)**. Intestinal organoids expressed the intestine‐specific TFs CDX2 and ASCL2 and showed polarisation of E‐cadherin and acetyl‐α‐tubulin **(c, d)** (maximum intensity projection of Z‐stack; scale bars 50 μm). qPCR analyses on bulk RNA from iPSCs, foregut spheroids and fully developed organoids showed timely expression of lung‐specific **(e)** and intestine‐specific **(f)** TFs. Results are expressed as mean (SD) of three independent experiments. Data were analysed with a Tukey‐corrected one‐way ANOVA. **P ≤ 0.01, ***P ≤ 0.001. Flow cytometry shows that both the organoids contain three main cell populations based on the expression of CD73 and EpCam **(g, h)**. Overall, organoids showed high expression of TLR4 and low expression of TLR2. Both in lung **(g)** and intestinal **(h)** organoids, the endothelial‐like CD73^+^ EpCam^−^ population showed higher expression of TLR2 and TLR4. Gated on singlets, live cells. Results of three independent experiments are shown. TLR expression was also measured at mRNA level. qPRC analyses demonstrate that both lung **(i)** and intestinal **(j)** organoids express different TLRs, especially TLR2 and TLR4. Finally, NF‐κB reporter organoids demonstrated NF‐κB activation following LPS triggering, suggesting that organoids possess functional TLRs **(k, l)**. Results are expressed as mean (SD) of three independent experiments. Data were analysed with an unpaired *t*‐test. **P ≤ 0.01, ***P ≤ 0.001.

We generated LOs and IOs from human‐induced pluripotent stem cells (iPSCs) following established protocols.^25^, ^26^ To validate the success of the differentiation protocols, we measured expression of mucosal tissue markers at the mRNA and protein levels. Immunofluorescent labelling revealed that LOs express the pulmonary transcription factor (TF) FOXJ1 (Figure 1a), while IOs express the intestinal TFs CDX2 and ASCL2 (Figure 1c and d), as previously described.^27^, ^28^ We confirmed these data by qPCR, showing that LOs express the lung TFs *FOXJ1* and *NKX2.1* (Figure 1e), while IOs express the intestine‐specific TFs *ASCL2* and *CDX2* (Figure 1f). Notably, these TFs were not expressed by iPSCs but became detectable during the foregut and hindgut spheroid stages, respectively (Figure 1e and f). Similar to primary tissues,^29^, ^30^ both LOs and IOs express E‐cadherin at adherent junctions (Figure 1b and c), exhibit tissue polarisation characterised by acetyl‐α‐tubulin labelling at the organoid apical side (Figure 1a and d) and are positive for phalloidin staining, suggesting the presence of ciliated cells facing the organoid lumen (Figure 1b). Finally, LOs but not IOs stain positively for mucin (Supplementary figure 1a).

The major advantage of using organoids as a model of the tissue microenvironment is their intrinsic complexity and cellular composition, which closely resembles that of primary tissues. As mucosal tissues comprise cells of epithelial and mesenchymal origin,^1^ we first confirmed the presence of EpCam‐1^+^ epithelial cells, as well as CD73^+^ mesenchymal cells, in both LOs and IOs by flow cytometry (Figure 1g and h). We next asked whether these cells expressed Toll‐like receptors (TLRs). qPCR analyses revealed detectable levels of a suite of TLRs across both organoid types (Figure 1i and j), with more abundant expression in IOs (Figure 1j) than in LOs (Figure 1i, Supplementary figure 1b), and TLR4 being the most highly expressed in both. Notably, the human iPSC line used for organoid development shows no TLR2 or TLR4 expression (Supplementary figure 1c). Flow cytometry analysis confirmed the surface expression of TLR4 on both epithelial and mesenchymal subsets of LOs (Figure 1g) and IOs (Figure 1h), while TLR2 surface expression was detected only in a subset of the endothelial and mesenchymal cells in both LOs (Figure 1g) and IOs (Figure 1h). Overall, LOs show higher expression of TLR2 and TLR4 (Supplementary figure 1d).

To assess whether the TLRs were capable of initiating downstream signalling following ligation, we developed an iPSC line harbouring an NF‐κB luciferase reporter and used it to differentiate lung and intestinal organoids. Exposure of these organoids to the TLR4 ligand lipopolysaccharide (LPS) induces significant luciferase activity in LOs (Figure 1k) and IOs (Figure 1l), demonstrating TLR4 functionality.

In summary, we demonstrated that both LOs and IOs express specific markers of lung and intestinal mucosal tissues and show signs of tissue polarity, a key property of mucosal tissues. Moreover, both organoid types express multiple TLRs, of which we confirmed functional activation of the TLR4‐NF‐κB axis following LPS stimulation. These results demonstrate that iPSC‐derived mucosal organoids can sense and respond to pathogen recognition receptor (PRR) ligands and thus provide a suitable *in vitro* model for the study of host–pathogen interactions.

### Mucosal tissue organoids express an inflammatory transcriptional signature upon PRR stimulation

**Figure 2:**
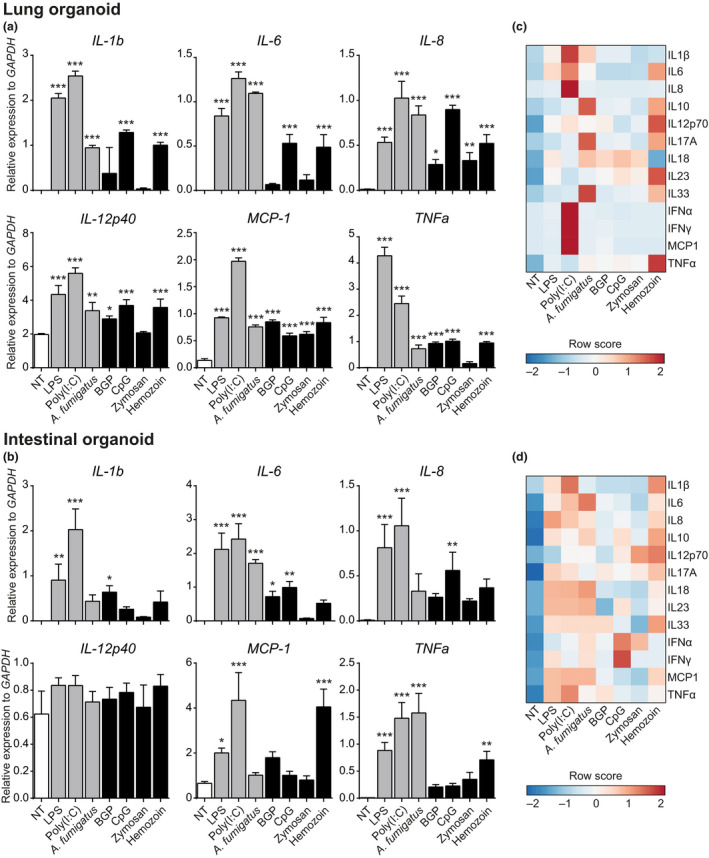
Lung and intestinal organoids respond to PRR ligands. Stimulation of lung **(a)** and intestinal **(b)** organoids with a variety of soluble and particulate PRR ligands induced the transcription of pro‐inflammatory cytokines. These results were confirmed at the protein level by cytometric bead immunoassay on lung **(c)** and intestinal **(d)** organoid supernatants after 24‐h stimulation. Results are expressed as mean (SD) of three independent experiments. Data were analysed against NT via a Dunnett‐corrected one‐way ANOVA. *P ≤ 0.05, **P ≤ 0.01, ***P ≤ 0.001.

To extend our previous findings on the functional activation of TLR4 signalling pathways, we stimulated the organoids with a set of purified PRR ligands (LPS, poly(I:C), CpG) and particulate preparations [heat‐killed *Aspergillus fumigatus*, β‐glucan particles (BGP), zymosan and *Plasmodium* spp. hemozoin]. PRR stimulation of both lung and intestinal organoids resulted in significant upregulation of expression of inflammatory cytokines including IL‐1β, IL‐6, IL‐8, TNF‐α and MCP‐1 (Figure 2a – LOs, Figure 2b – IOs). Most cytokines are expressed by both organoid types, though expression of IL‐12p40 and TNFα is higher in LOs (Figure 2a), while MCP‐1 and IL‐6 are more highly expressed in IOs (Figure 2b). While some ligands induce a common mRNA expression signature in the two organoid types, for example, *A. fumigatus* and CpG, others, such as hemozoin – a by‐product of RBC lysis from the malaria agent *Plasmodium* – strongly induce MCP‐1 expression in IOs but only poorly in LOs. We further validated and expanded our results using a bead array to measure protein expression of 13 human inflammatory cytokines: levels of IL‐1β, IL‐6 and MCP‐1 increase in both organoid types upon PRR stimulation (Figure 2c and d), while IL‐8, TNF‐α, INF‐α and IFN‐γ are expressed only by LOs (Figure 2c); and IOs specifically secrete IL‐10, IL‐23 and IL‐33 upon stimulation with LPS, poly(I:C), *A. fumigatus* and hemozoin (Figure 2d).

In summary, we showed that LOs and IOs respond to PRR stimulation (especially to poly(I:C), LPS and *A. fumigatus*) by expressing and releasing several cytokines. Of note, cytokine signatures differ between the two organoid types upon stimulation by the same PRR ligands.

**Figure 3:**
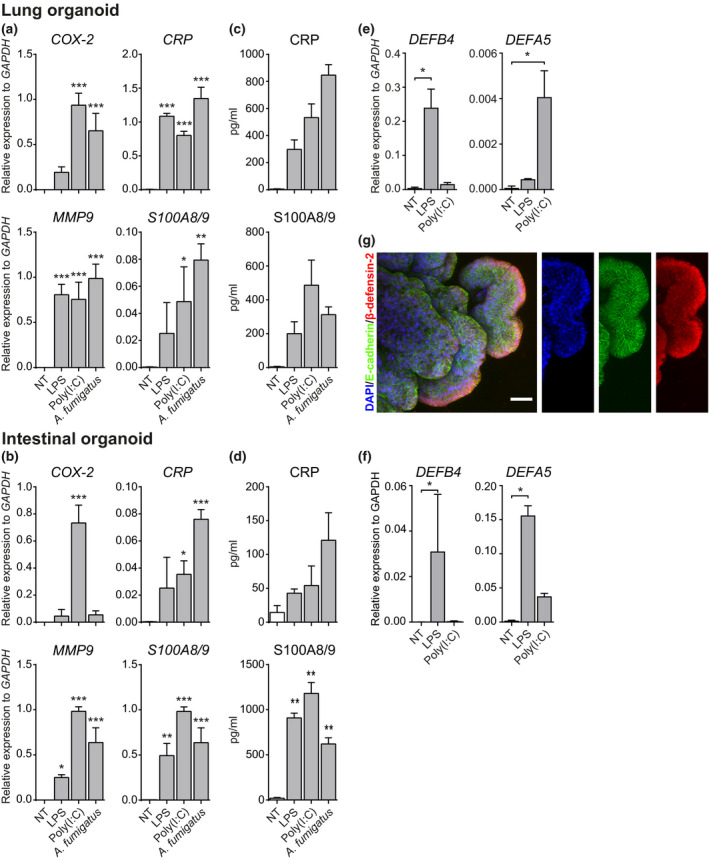
PRR ligands induce tissue‐specific inflammatory expression signatures in organoids. Triggering of lung **(a)** and intestinal **(b)** organoids with either LPS, poly(I:C) or *A. fumigatus* resulted in the expression of tissue‐specific inflammatory markers including COX‐2, CRP, MMP9 and S100A8/9. Results were confirmed at the protein level assessing CRP and S100A8/9 levels in lung **(c)** and intestinal **(d)** organoid supernatants by ELISA. qPCR analyses showed that the organoids differentially expressed genes encoding β‐defensin‐2 and β‐defensin‐5. While lung organoids mainly expressed β‐defensin‐2 **(e)**, intestinal organoids expressed higher levels of β‐defensin‐5 **(f)**. LPS and, to a lesser extent, poly(I:C) treatment induced upregulation of the transcripts encoding both the β‐defensins **(e, f)**. Immunofluorescent labelling confirmed the polarised expression of β‐defensin‐2 by lung organoids **(g)**. Results are expressed as the mean (SD) of three independent experiments. Data were analysed against NT via a Dunnett‐corrected one‐way ANOVA. *P ≤ 0.05, **P ≤ 0.01, ***P ≤ 0.001.

To further dissect the inflammatory environment induced by PRR stimulation on organoids, we measured mRNA expression levels of tissue inflammation markers such as the enzymes cyclooxygenase 2 (COX‐2) and matrix metalloproteinase 9 (MMP9), the acute‐phase protein C‐reactive protein (CRP) and the alarmin S100A8/9. LPS, poly(I:C) and *A. fumigatus* triggering induces MMP9 expression in both LOs (Figure 3a) and IOs (Figure 3b). In contrast, COX‐2 expression is induced by poly(I:C) and *A. fumigatus* in LOs (Figure 3a) but only by poly(I:C) in IOs (Figure 3b). We further checked for the expression and secretion of CRP in both LOs and IOs: although CRP transcripts are present in both organoid types after PRR triggering, LOs expressed significantly more mRNA and released higher levels of the protein (Figure 3a and c – LOs; Figure 3b and d – IOs). While S100A8/9 is also expressed and released following PRR triggering in LOs, it is more abundant in IOs (Figure 3c and d).

Additionally, we checked for the modulation of β‐defensins – antimicrobial peptides secreted by mucosal and epithelial tissues – in response to LPS and poly(I:C) triggering. LPS was the most potent inducer of β‐defensin expression in both organoid types (Figure 3e and f) although poly(I:C) also induced β‐defensin‐5 (DEFA5) expression in LOs (Figure 3e). mRNA levels of β‐defensin 2 (DEFB4) are higher in LOs (Figure 3e), while those of β‐defensin 5 are higher in IOs (Figure 3f). Furthermore, although basal mRNA levels are low, β‐defensin‐2 protein is detectable by immunolabelling in steady‐state LOs (Figure 3g).

Overall, these results indicate that LOs and IOs upregulate expression of inflammatory mediators including MMP9, COX‐2, S100A8/9 and CRP during exposure to PRR ligands. Moreover, both organoid types differentially modulate expression of β‐defensins both in the steady state and during infection.

### Global gene expression analysis of PRR‐stimulated organoids confirms tissue‐specific signatures and immune‐competent microenvironment

**Figure 4:**
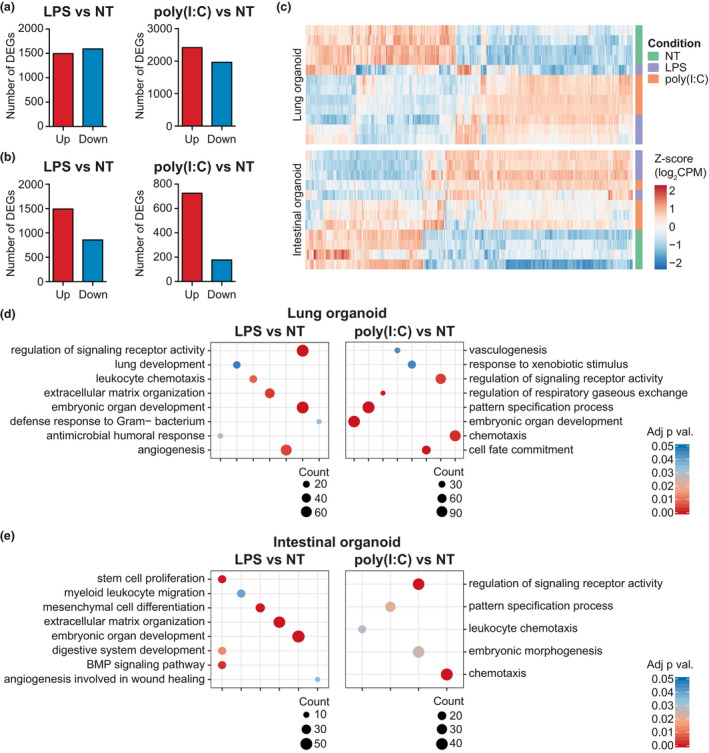
PRR ligands induce modulation of pathways involved in organoid development and immune cell recruitment. Bulk RNA‐seq analysis of LPS‐ and poly(I:C)‐treated organoids revealed regulation of several genes (fold change ≥ ±2 and adj. *P*‐value ≤ 0.05) **(a)**, lung organoids; **(b)**, intestinal organoids). Unsupervised hierarchical clustering of differentially expressed genes (DEGs) from RNA‐seq data shows consistent clustering among the samples **(c)**. Gene ontology analyses on biological process (BP) of the upregulated genes demonstrate that both LPS and poly I:C triggering activated pathways involved in leucocyte chemotaxis as well as pathways involved in embryonic organ development, vasculogenesis and extracellular matrix organisation **(d, e)**.

In order to obtain broader information about the organoids' microenvironment and their specific tissue‐like phenotype, we performed bulk RNA sequencing on organoids stimulated with the strongest triggers of inflammatory cytokine production: LPS and poly(I:C). Stimulated LOs and IOs exhibit distinct gene expression signatures (Figure 4a–c). Gene ontology analyses on upregulated genes demonstrated that both LPS and poly(I:C) induce pathways involved in leucocyte chemotaxis and immune antimicrobial responses (Figure 4d and e). Interestingly, further dissection of the genes driving leucocyte chemotaxis pathways showed that LPS and poly(I:C) treatment induces increased expression of distinct chemokines by the two organoids (Supplementary figure 2a). As an example, LOs upregulate CCL11 in response to LPS, but CCL3L1 and CXCL13 in response to poly(I:C). On the one side, LPS treatment of IOs results in the expression of CXCL1/3/5/12/14, while poly(I:C) triggering leads to increased expression of CXCL6/8/20 (Supplementary figure 2a).

Lipopolysaccharide triggering induces upregulation of expression of genes involved in extracellular matrix organisation in both organoid types. Both the PRR triggers activate pathways involved in embryonic organ development and pattern specification processes, suggesting a key role of PRR triggering during stem cell development. Furthermore, activation of these developmental pathways often correlates with activation of lung and intestinal specification signatures (as ‘lung development’, ‘digestive system development’).

To understand how the organoid types responded differently to LPS or poly(I:C) triggering, we compared the DEGs of IOs and LOs against the same trigger and then subjected them to gene ontology analysis (Supplementary figure 2b and c). IOs respond to LPS by regulating the expression of genes involved in acute inflammatory responses, and the response to bacteria and wound healing (Supplementary figure 2b), while LOs are more responsive to poly(I:C) stimulation, regulating genes involved in TLR signalling, type I IFN signalling and response to viruses (Supplementary figure 2c).

Taken together, the global RNA sequencing of lung and intestinal organoids upon PRR stimulation recapitulates the inflammatory phenotype previously observed using qPCR and protein assays and suggested that PRR signalling might be crucial for stem cell development.

### Mucosal tissue organoids are able to interact with human monocytes

Since our data suggested that organoids might be able to induce immune cell chemotaxis – especially through MCP1 after PRR ligation – we set up a coculture of steady‐state LOs and IOs with untouched human peripheral blood monocytes.

**Figure 5:**
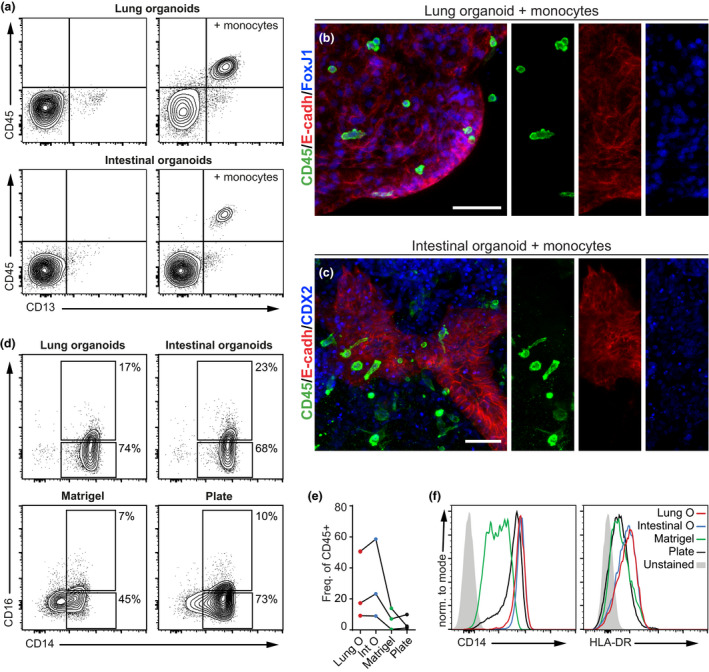
Human monocytes are recruited to organoids and acquire an intermediate‐like phenotype. Human monocytes were mixed with mature organoids and cocultured in Matrigel for 72 h. Prior to coculture with monocytes, organoids were devoid of CD45^+^ hematopoietic cells, as assessed by flow cytometry, while after coculture, a population of CD45^+^ CD13^+^ cells was associated with both organoid types **(a)**. Gated on singlets, live cells. Confocal analysis confirms that cocultured CD45^+^ monocytes interact closely with lung **(b)** and intestinal organoids **(c)**, showing an elongated morphology typical of tissue‐infiltrating monocytes (maximum intensity projection, scale bar 100 μm). Monocytes cocultured with organoids showed a more intermediate‐like surface marker phenotype than monocytes cultured on plastic or alone in Matrigel, as indicated by the expansion of the CD14^+^ CD16^+^ fraction, and the higher expression of CD14 and HLA‐DR **(d–f)**. Gated on FSC/SSC, singlets, live cells, CD45^+^. Results of three independent experiments are shown.

We began by confirming that both LOs and IOs were devoid of CD45^+^ immune cells of hematopoietic origin (Figure 5a); we then generated Matrigel cocultures containing organoids and purified human monocytes and observed them after 72 h. Monocytes had migrated to the organoids (Figure 5a) and were tightly associated with the basal cell layer (Figure 5b and c), where they had adopted an elongated shape and begun to extend cellular protrusions between the E‐cadherin+ epithelial cells (Figure 5b and c; Supplementary video 1).

Compared to monocytes cultured on plastic or alone in Matrigel, monocytes interacting with organoids alter their surface marker phenotype to resemble ‘intermediate monocytes’, expressing increased levels of CD14 and CD16 (Figure 5d), resulting in increased frequencies of double‐positive CD14^+^ CD16^+^ monocytes than control cultures (Figure 5e). These monocytes also express higher levels of HLA‐DR than controls (Figure 5f).

These results show that human monocytes migrate towards organoids and interact at their basal side. Through this interaction, monocytes develop an ‘intermediate’ phenotype expressing higher levels of CD14 and CD16 and elevated HLA‐DR. Taken together, this demonstrates that human iPSC‐derived mucosal organoids provide a suitable culture environment for maintaining and dynamically differentiating immune cells such as monocytes.

## Discussion

Mucosal tissue interfaces play a key role in the maintenance of organismal homeostasis by mediating communication between the external space and the cells, while regulating microbiota sampling. However, dissecting the molecular mechanisms that orchestrate mucosal tissue microenvironmental responses remains challenging, because of the technical and biological limitations of current experimental approaches. Here, we propose and test a strategy to study mucosal tissue immunocompetence and responses to inflammatory stimuli using human iPSC‐derived 3D tissue organoids.

Since PRRs play a key role during infection, we aimed to describe their presence and activity in our 3D systems. Both LOs and IOs expressed different TLRs, and we directly demonstrated the functionality of TLR4, showing NF‐κB activation following LPS triggering. TLR2 and TLR4 were mostly expressed by a mesenchymal/endothelial CD73^+^ EpCam^−^ population, suggesting that endothelial and epithelial cells might play distinct roles in the control of tissue inflammation. Studies in TLR reporter mice show that intestinal epithelial cells (IECs) in adult murine small intestine express low levels of different TLRs while colonic IECs express them at a higher level.^31^ Interestingly, in these studies the strongest TLR signal came from EpCam^−^ cells underlying the IEC layer; however, it is impossible to dissect whether these cells were of endothelial or hematopoietic origin, as the mice used for the study were not depleted of immune cells.

We also found that stimulating organoids with a panel of TLR ligands induced the release of several cytokines and chemokines, even in the absence of immune cells. Our findings corroborate the observation of Hibiya *et al*.,^32^ who reported upregulation of several NF‐κB‐controlled immune mediators (including IL‐8) upon PRR stimulation of colon organoids. Similarly, murine colonic, but not small intestine, organoids responded to a panel of TLR ligands and pro‐inflammatory cytokines by expressing cytokines and chemokines including *Tnf*, *Cxcl10* and *Il1b*.^31^ Global gene expression analysis of bacterially infected human intestinal organoids also revealed enrichment in processes including inflammatory response, epithelial development, and response to LPS and chemotaxis,^23^, ^24^ which were similarly significantly enhanced in our study.

Notably, our LOs specifically responded to poly(I:C) triggering by releasing IFN‐α and IFN‐γ, and regulating expression of genes involved in type I interferon pathways. These results are in accordance with clinical data showing the importance of IFN responses in the control of pulmonary viral infections^33^ and reveal the potential for LOs in particular to model *in vivo* responses.

We further analysed the expression of COX‐2 and MMP9, which confirmed that an inflammatory milieu was induced by PRR triggering in both IOs and LOs.^34^, ^35^ Moreover, we detected differential expression and release of two other inflammatory markers: CRP and S100A8/9. CRP was strongly expressed and released only by LOs, consistent with previous findings of CRP release in the human respiratory tract.^36^ Similarly, other studies using human and animal models show that CRP expression is much higher in LOs than in IOs.^37^, ^38^ On the contrary, the S100A8/9 was more highly expressed in IOs following PRR triggering. This alarmin is released during infection and plays a role in modulating the inflammatory response by stimulating immune cell recruitment and inducing cytokine secretion.^39^

Additionally, we showed that organoids differentially expressed genes encoding antimicrobial β‐defensins: while IOs expressed higher levels of β‐defensin‐5, LOs expressed higher levels of β‐defensin‐2 (hBD‐2). Moreover, LPS and poly(I:C) triggering upregulated their expression in both organoid types. These results are supported by the fact that hBD‐2 was induced by the causative agent of Legionnaires' disease, *Legionella pneumophila*, in a TLR2‐TLR5‐NF‐κB fashion.^40^

Although we mainly used purified microbial ligands, we believe that this system might also be useful to study the interactions between tissues and living microorganisms (both pathogens and resident microbiota). However, care should be taken because these microorganisms might be difficult to coculture in the absence of specific nutrition (e.g. in the gastrointestinal tract) and of physiological O_2_ concentrations. Moreover, it would be quite challenging to precisely titrate the number of inoculated microorganisms and control their numbers during the coculture.

Global expression analysis of LPS‐ and poly(I:C)‐stimulated organoids suggested alterations in tissue developmental signatures, showing activation of several pathways involved in embryonic organ development, extracellular matrix organisation and pattern specification processes. The role of cytokine and PRR stimulation in organoid differentiation was reported earlier.^32^ For example, IL‐13 treatment of 3D airway epithelium demonstrated that inflammatory signalling promotes the development of goblet cells in human 3D broncospheres via Notch signalling, thus increasing mucus production.^41^ Nigro *et al*. investigated the potential of microbial compounds to stimulate intestinal stem cells within organoids; they found that Nod receptor ligands were sufficient to promote IO survival and stem cell cytoprotection. Furthermore, a similar study by Davies *et al*.^42^ implied that poly(I:C) decreased gene expression associated with epithelial differentiation and lysozyme‐positive stem cells in gut and colon organoids, while increasing the expression of inflammatory genes. Although being a matter of strong debate, recent studies suggest the existence of a ‘placental microbiota’ and thus a maternal–foetal transfer of commensal microbiota.^43^ Nonetheless, it was recently shown that maternal immunoglobulins were able to transfer microbial molecules to the embryo, in the absence of a placental microbiota. Transient microbial exposure of germ‐free animals during pregnancy caused changes in the transcriptome of pups' small intestines, upregulating genes involved in epithelial cell division and differentiation, metabolism of xenobiotics and mononuclear cell function and recruitment.^44^ Overall, these results are in concert with our data and suggest an important role of PRR signalling during stem cell and embryonic organ development.

One of the remaining challenges of immunology research is to dissect the origin and exact functions of immune cells infiltrating the tissues. The phenotype of organ‐infiltrating myeloid cells is extremely dynamic and differs from organ to organ, such that even the identification of specific cell subsets within genetic models *in vivo* remains challenging.^45^, ^46^ Previous studies show the potential of monocytes to migrate into mucosal tissues and repopulate tissue‐resident macrophages during inflammation.^47^ Here, we demonstrate that primary human monocytes migrate towards mucosal organoids and interact at their basal side in the absence of exogenous PRR stimulation, consistent with monocytes' nature as steady‐state surveillants. Our coculture data show that monocytes can survive in coculture with the organoids and express higher levels of CD14, CD16 and HLA‐DR in comparison with cells grown on plastic or in Matrigel alone. Our results are in agreement with the few immune cell‐epithelial studies published by others: in an enteroid–macrophage coculture, Noel *et al*.^48^ observed morphological changes and the induction of cytokine production by macrophages, including of TGF‐β 1, IL‐8, IFN‐γ and IL‐6. However, this model used a porous membrane to sustain the development of the intestinal epithelium, thus significantly limiting the surface available for cell–cell interaction. Another 3D intestinal coculture model between immortalised macrophages and colon carcinoma‐derived spheroids demonstrated an important role of macrophages in the control of *Salmonella* infections.^49^ Furthermore, Nozaki *et al*.^50^ demonstrated that intraepithelial lymphocytes expand during coculture with intestinal epithelial organoids in the presence of IL‐2, IL‐7 and IL‐15 and also migrate towards the organoids over a period of 2 weeks.

Taken together, we demonstrate that iPSC‐derived lung and intestinal tissue organoids model the mucosal tissue microenvironment and respond rapidly and appropriately to stimulation with a range of biologically relevant PRR ligands by producing a suite of pro‐inflammatory mediators, independent of hematopoietic immune cells. Furthermore, these organoids are capable of attracting and supporting the differentiation of human monocytes in the steady state, thus providing a novel tool to study the developmental trajectories of immune cells within the tissue microenvironment.

## Methods

### Human‐induced pluripotent stem cell maintenance

Undifferentiated human‐induced pluripotent stem cells (iPSCs), DF19‐9‐7T (WiCell, Madison, WI, USA^51^), were maintained in mTESR‐1 medium (Stemcell Technologies, Vancouver, BC, Canada) on hES‐qualified Matrigel‐coated tissue culture plates, according to the manufacturer's instructions. Cells were fed daily by replacing all the medium and were passaged using collagenase (iv) at a concentration of 2 mg mL^−1^ when reaching approximately 80% confluence.

### Lung organoid (LO) differentiation

Induced pluripotent stem cells were differentiated into 3D lung organoids by a three‐step differentiation process (as described in De Luca *et al*.^52^ adapted from Dye *et al*.^25^). In brief, iPSCs reaching ~60–70% confluence were induced into endoderm differentiation using RPMI1640 supplemented with penicillin (500 U mL^−1^), streptomycin (500 U mL^−1^) and activin A (100 ng mL^−1^) (Stemcell Technologies) and with endotoxin‐free Hyclone‐defined FBS (GE Healthcare Bio‐Sciences, Pittsburgh, PA, USA) at 0% (day 1), 0.2% (day 2) and 2% (day 3) to develop definitive endoderm. The endodermal layer was further induced to foregut differentiation by changing the medium daily [advanced DMEM/F12 supplemented with N2 supplement (1× final dilution), B27 supplement (1× final dilution), HEPES (15 mm), glutamine (2.5 mm), penicillin (500 U mL^−1^), streptomycin (500 U mL^−1^), Noggin (200 ng mL^−1^), SB431542 (10 μm), SAG (1 μm), CHIR99021 (2 μm) and FGF4 (500 ng mL^−1^)] for 5 days. 3D spheroid structures formed at day 5 of differentiation were collected under a microscope, and 30 spheroids were pooled into a 1.5‐mL microcentrifuge tube with 25 μL of medium. The medium‐spheroid suspension was mixed with an equal volume of Matrigel, placed like a bead over Nunc Sphera 24‐well plates (Thermo Fisher Scientific, Waltham, MA, USA) and fed with LO medium [as for foregut differentiation medium with addition of FGF10 (500 ng mL^−1^) and 1% FBS] every 3 days. In about 20 days, 3D organoids with the features of human lung tissues were obtained and were then split and maintained in LO medium. All growth factors and small molecules were from R&D Systems, Minneapolis, MN, USA; RPMI, advanced DMEM/F12, penicillin, streptomycin, Hyclone‐defined FBS, N2 supplement, B27 supplement, HEPES and glutamine were from Thermo Fisher Scientific.

### Intestinal organoid (IO) differentiation

Induced pluripotent stem cells were differentiated into 3D intestinal organoids by a three‐step differentiation process, similar to the LO differentiation and as described in McCracken *et al*.^26^ iPSCs were differentiated to definitive endoderm as described in the LO method. The endodermal layer was fed with hindgut differentiation medium daily [RPMI1640 supplemented with HEPES (15 mm), glutamine (2.5 mm), penicillin (500 U mL^−1^), streptomycin (500 U mL^−1^), (FGF4 200 ng mL^−1^) and WNT3a (500 ng mL^−1^)] for 4 days. On day 4, 3D spheroids were collected and embedded in Matrigel as described above. Matrigel‐embedded spheroids were fed with IO medium weekly [advanced DMEM/F12 supplemented with B27 (1× final dilution), HEPES (15 mm), glutamine (2.5 mm), penicillin (500 U mL^−1^), streptomycin (500 U mL^−1^), Noggin (100 ng mL^−1^), R‐spondin1 (200 ng mL^−1^), EGF (100 ng mL^−1^) and 1% Hyclone‐defined FBS]. After about 15 days of culture, 3D organoids exhibiting features of human intestinal tissues were obtained and were split and maintained in IO medium. All growth factors and small molecules were from R&D Systems.

### Organoid stimulation

Fully differentiated organoids were stimulated for 24 h with a panel of pattern recognition receptor ligands: 10 μg mL^−1^ LPS‐EB, 20 μg mL^−1^ HMW poly(I:C), 5 μg mL^−1^ BGP, 5 μg mL^−1^ CpG (ODN2216) and 5 μg mL^−1^ Zymosan (all from Invivogen, San Diego, CA, USA). *P. falciparum* natural hemozoin (nHZ) was collected from synchronised 3D7 parasite cultures from the 10%/40% interphase of a 6% mannitol‐containing Percoll gradient and washed repeatedly with 10 mm phosphate buffer.

Particulate ligands (such as heat‐inactivated *A. fumigatus* and hemozoin) were microinjected into the organoid luminal cavity using a XenoWorks Digital Microinjector (Sutter Instruments, Novato, CA, USA) at an estimated concentration of 10 particles per organoid cell.

### Immunofluorescence

Organoids were fixed for 20 min at RT with PFA 4% (Chem Cruz Biochemicals, Dallas, TX, USA), washed three times with PBS, permeabilised for 15′ with PBS + 0.5% Triton X‐100 and washed three times with IF buffer (PBS + 0.2% Triton X‐100 + 0.05% Tween‐20). Nonspecific binding sites were blocked by incubating for 1 h at RT with 2.5% BSA in IF buffer. Organoids were labelled with primary antibodies in IF buffer + 1% BSA overnight at 4°C (clones and dilutions in Supplementary table 1), then washed three times in IF buffer and labelled with secondary antibodies and phalloidin in IF buffer + 1% BSA for 2 h at RT. Finally, organoids were washed three times in IF buffer, counterstained for 10′ with DAPI in IF buffer, washed again and immediately imaged with a Zeiss LSM 780 confocal microscope at 10× magnification. Image processing was performed using ImageJ2.^53^ The Supplementary video 1 was prepared with Bitplane Imaris v9.2.1 (Belfast, UK).

### Flow cytometry

Organoids were retrieved from Matrigel by dissolving the matrix with cold PBS. After extensive washing to remove Matrigel debris, organoids were incubated in trypsin–EDTA (Thermo Fisher Scientific) for 10′ at 37°C, filtered at 70 μm to obtain single‐cell suspensions and centrifuged for 10′ at 300 *g*. Single cells were then labelled with fluorochrome‐conjugated antibodies for 30′ on ice (clones, conjugations and dilutions in Supplementary table 2), washed and fluorescence‐acquired on a spectral Sony SA3800 flow cytometer (Sony Biotechnology, San Jose, CA, USA). If monocytes were added to the coculture, organoids were extensively washed with cold PBS and allowed to settle by gravity before trypsinisation, in order to remove the monocytes not bound to the organoids. Control monocytes incubated in Matrigel alone or on cell‐culture plastic were processed in the same way before labelling. Results were analysed with FlowJo v10 (BD Life Sciences, Ashland, OR, USA).

### Gene expression assay

RNA was isolated by column separation using a RNeasy Plus Micro Kit with gDNA elimination (Qiagen, Hilden, Germany). All qPCR assays were run using TaqMan probes and TaqMan™ Gene Expression Master Mix (Thermo Fisher Scientific) in a Roche Lightcycler 480. TaqMan^®^ Gene Expression Assay probes used for qPCR experiments are listed in Supplementary table 3.

### RNA sequencing

Lung and intestinal organoids derived from four independent differentiations were stimulated with either 10 μg mL^−1^ LPS, 20 μg mL^−1^ poly(I:C) or left untreated. Total RNA was isolated from organoids using RNeasy kit (Qiagen), according to the manufacturer's protocol. RNA quality was assessed with Bioanalyzer2100 RNA Nano 6000 chips (Agilent Technologies, Santa Clara, CA, USA), and samples with RIN > 9 were used for sequencing. An Illumina sequencing library was prepared using the NEBNext Ultra II Directional RNA Library Prep Kit (New England Biolabs, Ipswich, MA, USA) following the manufacturer's protocols. Briefly, total RNA was used for polyA enrichment, then fragmented and transcribed into cDNA. Following universal adapter ligation, samples were barcoded using NEB dual indexing primers and pooled equimolarly after picogreen quantitation. Sample pool was sequenced using Nextseq 550 sequencer (Illumina, San Diego, CA, USA) using a 75 cycles high output cartridge.

Raw reads were mapped to the reference genome (genome version: Ensembl GRCh38), counted and summarised to genes (gene annotation: Ensembl v94).

Differentially expressed genes (DEGs) were identified using the limma‐trend approach after data filtration and normalisation.^54^ Significantly DEGs (identified as fold change ≥ ±2 and adj. *P* value ≤ 0.05) were used as input for Gene Ontology analyses using clusterProfiler.^55^ All the analyses were performed in R environment.

### Bead array cytokine detection

Supernatants from PRR‐stimulated lung and intestinal organoids were used to determine the cytokine expression profiles using the LEGENDplex™ Human Inflammation Panel [13‐plex including IL‐1β, IFN‐α2, IFN‐γ, TNF‐α, MCP‐1 (CCL2), IL‐6, IL‐8 (CXCL8), IL‐10, IL‐12p70, IL‐17A, IL‐18, IL‐23 and IL‐33] cytometric bead array (BioLegend, San Diego, CA, USA), according to the manufacturer's protocol.

### ELISA

Commercial DuoSet ELISA development kits (R&D Systems) were used for detection of CRP and S100A8/9 in supernatants and were performed in accordance with manufacturer's instructions.

### NF‐kB‐luciferase reporter iPSC line and luminescence assay

Induced pluripotent stem cells maintained in mTESR‐1 medium as described above were transfected using Cignal Lenti NF‐κB(luc) Reporter (Qiagen) according to the manufacturer's protocol and subsequently selected with 1 μg mL^−1^ puromycin (Santa Cruz Biotechnology, Dallas, TX, USA), to obtain a homogeneous population carrying the reporter. Organoids were developed from the NF‐kB reporter iPSCs as above and then stimulated with LPS (10 μg mL^−1^) for 18 h. Luciferase activity was detected using the ONE‐Glo Luciferase Assay System (Promega, Madison, WI, USA) in a microplate luminometer (Berthold Technologies, Bad Wildbad, Germany).

### Monocyte–organoid cocultures

Monocytes were isolated from fresh, healthy buffy coats (Department of Transfusion & Tissue Medicine of the Brno University Hospital) using the RosetteSep Human Monocyte Enrichment Cocktail (Stemcell Technologies) by gradient centrifugation on Lymphoprep (1.077 g mL^−1^, Stemcell Technologies). Monocyte purity was assessed by flow cytometry after labelling for CD14. Typical purity was ~90% (Supplementary figure 3a). Monocytes were mixed and embedded in Matrigel together with LOs and IOs and maintained for 72 h in DMEM/F12 medium at 37°C before immunolabelling for microscopy or flow cytometry.

### Statistical analyses

Statistical analyses were performed with GraphPad Prism 8 (GraphPad Software, San Diego, CA, USA). Unless otherwise indicated, results are expressed as mean (SD) of at least three independent experiments. Data were tested for normality before parametric or nonparametric statistical tests were applied. Statistical tests used for the specific analyses are indicated in each figure caption.

## Conflict of interest

The authors declare no conflict of interest.

## Author contribution

**Shyam Sushama Jose:** Conceptualization; Data curation; Formal analysis; Investigation; Methodology; Validation; Visualization; Writing‐original draft. **Marco De Zuani:** Conceptualization; Data curation; Formal analysis; Investigation; Software; Validation; Visualization; Writing‐original draft. **Federico Tidu:** Data curation; Investigation. **Marcela Hortová Kohoutková:** Data curation; Investigation. **Lucia Pazzagli:** Data curation; Investigation. **Giancarlo Forte:** Funding acquisition; Resources; Supervision. **Roberta Spaccapelo:** Resources; Supervision. **Teresa Zelante:** Funding acquisition; Supervision. **Jan Frič:** Conceptualization; Funding acquisition; Project administration; Resources; Supervision; Writing‐review & editing.

## Supporting information

Supplementary Material
