# Supplementary material for: Comparison of two human organoid models of lung and intestinal inflammation reveals Toll‐like receptor signalling activation and monocyte recruitment
Source: Clin Transl Immunology. 2020 May 5;9(5):e1131. doi: 10.1002/cti2.1131 (PMC7200218; doi:10.1002/cti2.1131)
Supplement: Supplementary file 1 — Supplementary Material [file CTI2-9-e1131-s001.pdf]

Comparison of two human organoid models of lung and intestinal inflammation reveals Toll-like receptor signalling activation and monocyte recruitment.

Supplementary material

Authors and affiliations: Shyam Sushama Jose[1#], Marco De Zuani[1#], Federico Tidu[1,2], Marcela Hortová Kohoutková[1], Lucia Pazzagli[3], Giancarlo Forte[1], Roberta Spaccapelo[3], Teresa Zelante[3], Jan Frič[1,4*]

[1] International Clinical Research Center, St. Anne's University Hospital Brno, Brno, Czech Republic

[2] Department of Biology, Faculty of Medicine, Masaryk University, Czech Republic

[3] Department of Experimental Medicine and University Research Center for Functional Genomic (C.U.R.Ge.F), University of Perugia, Perugia, Italy.

[4] Institute of Hematology and Blood Transfusion, Prague, Czech Republic

[#] The authors contributed equally

*Corresponding author

Corresponding address: Jan Frič, FNUSA-ICRC, Pekařska 53, Brno 656 91, jan.fric@fnusa.cz / Tel: +420 511 158 279

Supplementary figure 1

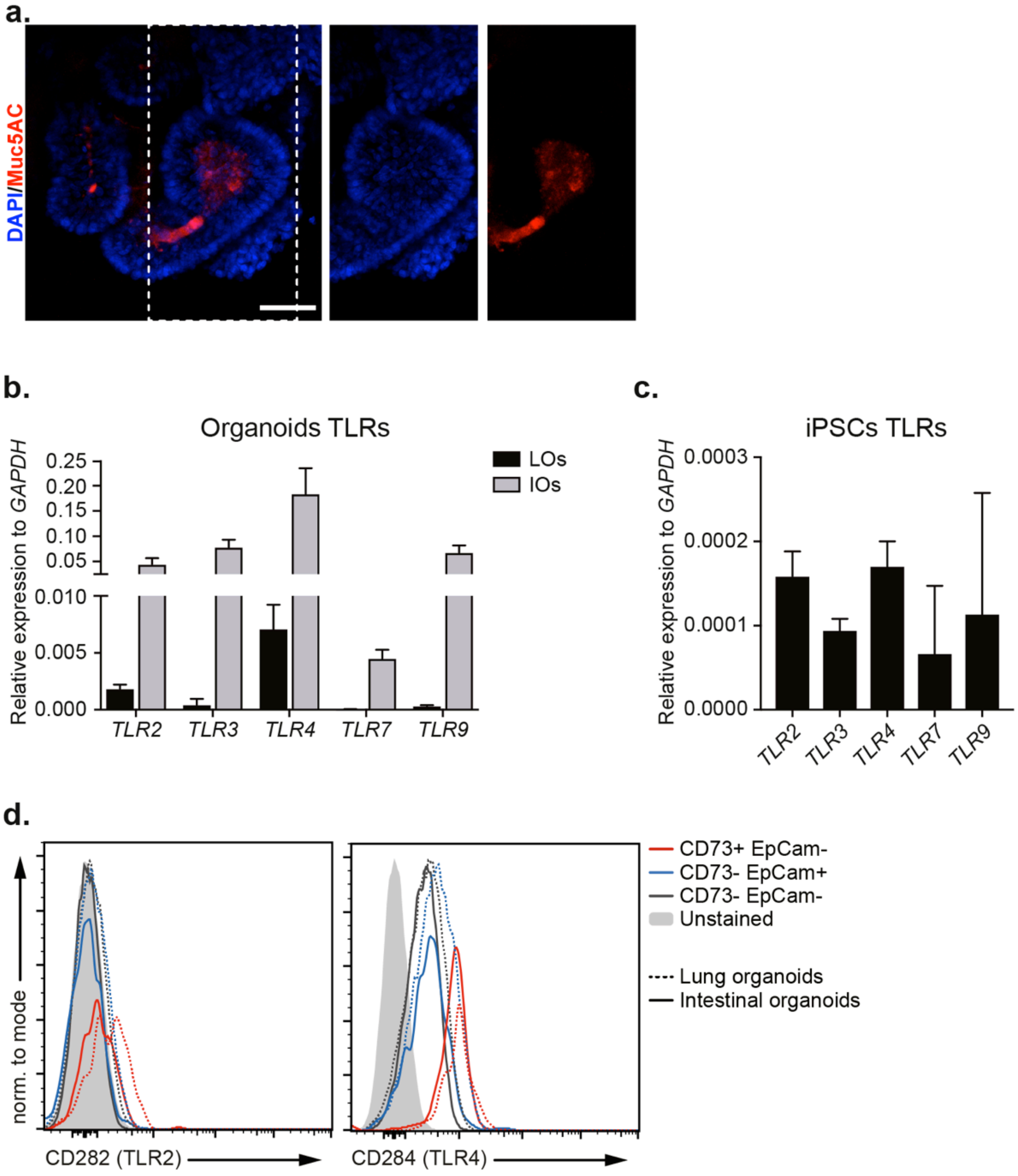

Supplementary figure 1: Organoid characterization. (a) Immunofluorescent labelling of Muc5AC shows the presence of mucins in the lumen of lung organoids. Maximum intensity projection of z-stack. Scale bar = 50μm. **(b)** qPCR analyses on iPSC show that these cells express very low levels of TLRs transcript before differentiation into organoids. **(c)** Flow cytometric analyses shows that both organoids express TLR2 and TLR4, and that LOs express higher levels of both TLRs. Dashed lines= lung organoids, filled lines= Intestinal organoids. Representative experiment of three replicates.

Supplementary figure 2

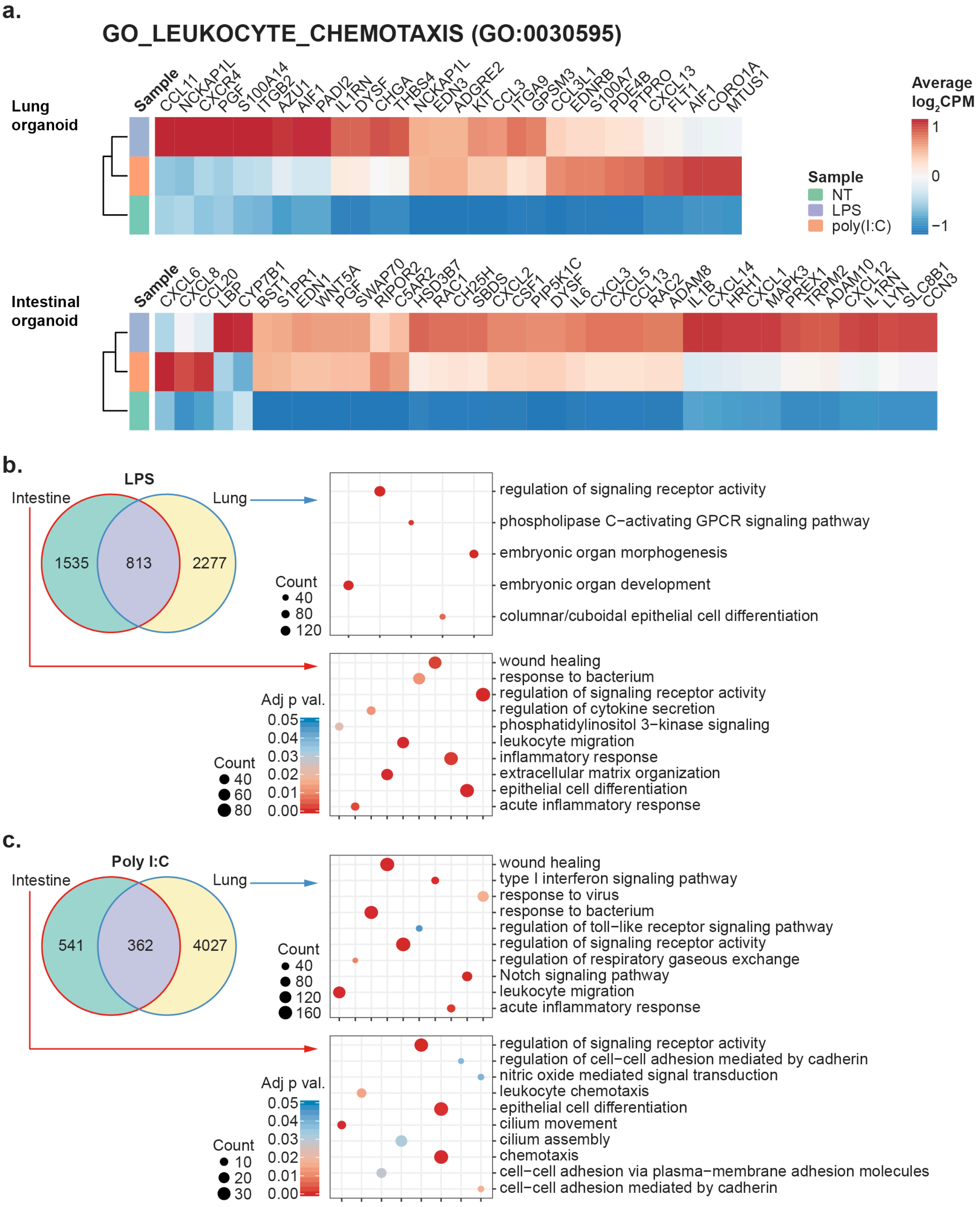

Supplementary figure 2: Lung and intestinal organoids shows differential response to LPS and poly(I:C). Dissection of the significantly up-regulated genes in response to LPS and poly(I:C) shows that these treatments differentially regulate genes involved in leukocyte

chemotaxis **(a)**. Venn plots built with the differentially expressed genes of stimulated lung and intestinal organoids shows that the two organoid models regulate different pathways against the same agonist. Intestinal organoids respond better to LPS stimulation, regulating pathways involved in acute inflammatory response, response to bacterium and wound healing **(b)**. On the contrary, poly(I:C) stimulation induces a more profound response in lung organoids, which regulate IFN signalling pathways and acute inflammatory responses **(c)**.

Supplementary figure 3

a.

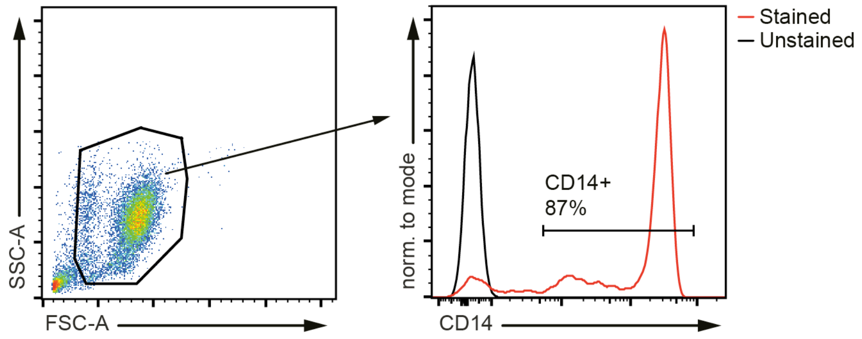

Supplementary figure 3: Monocyte purity. (a) Human monocytes were negatively isolated from fresh buffy coat. Purity was assessed by flow cytometry after staining for CD14. Representative results of 3 independent experiments are shown.

Supplementary video: Monocytes and lung organoids tightly interact at the basal layer. 3D- and volume representation of confocal Z-stack demonstrate that monocytes tightly associate with the organoid basal cell layer during the coculture, showing the presence of protrusions between the E-cadherin+ cells. Blue=DAPI, green=CD45, magenta=E-cadherin.

Supplementary table 1. List and details of antibodies used for immunofluorescent staining.

Antibody	Conjugation	Vendor	Clone	Catalogue #	Dilution
CD45	Unconjugated	Invitrogen	YAML501.4	MA517687	1:100
FoxJ1	Unconjugated	eBioscience	2A5	14-9965-82	1:100
E-cadherin	Unconjugated	Cell Signaling Technology	24E10	3195S	1:200
CDX2	Unconjugated	Invitrogen	ZC007	397800	1:100
Beta-2 defensin	Unconjugated	Invitrogen	-	PA5-47388	1:100
ASCL2	Unconjugated	Invitrogen	-	PA5-47852	1:50
Phalloidin	AlexaFluor 647	Invitrogen	-	A22287	1:200
Donkey anti-rat IgG	AlexaFluor 488	Invitrogen	-	A21208	1:500
Donkey anti-mouse IgG	AlexaFluor 555	Invitrogen	-	A31570	1:500
Donkey anti-rabbit IgG	AlexaFluor 647	Invitrogen	-	A31573	1:500
Donkey anti-mouse IgG	AlexaFluor 488	Invitrogen	-	A21202	1:500
Donkey anti-	AlexaFluor	Invitrogen	-	A31572	1:500

rabbit IgG	555				
Donkey anti-goat IgG	AlexaFluor 546	Invitrogen	-	A11056	1:500
Donkey anti-sheep IgG	AlexaFluor 555	Invitrogen	-	A21436	1:500

Supplementary table 2: List and details of antibodies used for flow cytometry experiments.

Antibody	Conjugation	Vendor	Clone	Catalogue #	Dilution
CD14	PE	BioLegend	63D3	367104	1:100
CD16	PE/Cy5	BioLegend	3G8	302010	1:100
HLA-DR	PE/dazzle 594	BioLegend	L243	307653	1:100
CD45	PacificBlue	BioLegend	2D1	368540	1:100
CD86	BV421	BioLegend	IT2.2	305426	1:100
CD68	FITC	BioLegend	Y1/82A	333806	1:100
CD184	PE/Cy7	eBioscence	12G5	25-9999-42	1:100
CD73	eFluor 450	eBioscience	AD2	48-0739-42	1:100
CD326 (EpCam)	Biotin	eBioscience	1B7	13-9326-82	1:100
Streptavidin	BV650	BioLegend	-	405231	1:200
CD282 (TLR2)	AlexaFluor 488	BioLegend	TL2.1	309712	1:100
CD284 (TLR4)	PE	eBioscience	HTA125	12-9917-42	1:100

Supplementary Table 3: List of TaqMan® Gene Expression Assay probes used for qPCR experiments.

**Gene ID**	**TaqMan Probe Id (Cat: 4331182)**
ASCL2	Hs00270888_s1
CDX2	Hs01078080_m1
LGR5	Hs00969422_m1
MCP-1	Hs00234140_m1
IL-8	Hs00174103_m1
IL-1b	Hs01555410_m1
IL-6	Hs00174131_m1
IL-12p40	Hs01011518_m1
TNFa	Hs00174128_m1
TLR2	Hs00610101_m1
TLR3	Hs01551078_m1
TLR4	Hs00152939_m1
TLR7	Hs00152971_m1
TLR9	Hs00152973_m1
MMP9	Hs00957562_m1
COX-2	Hs00153133_m1

S100A8	Hs00374263_m1
CRP	Hs00265044_m1
FOXJ1	Hs00230964_m1
NKX2.1	Hs00968940_m1
DEFB4	Hs00823638_m1
DEFB5	Hs00845803_g1
